# Acute Effects of Different Exercise Intensities on Executive Function and Oculomotor Performance in Middle-Aged and Older Adults: Moderate-Intensity Continuous Exercise vs. High-Intensity Interval Exercise

**DOI:** 10.3389/fnagi.2021.743479

**Published:** 2021-10-13

**Authors:** Chia-Liang Tsai, Yu-Chuan Chang, Chien-Yu Pan, Tsai-Chiao Wang, Jozef Ukropec, Barbara Ukropcová

**Affiliations:** ^1^Institute of Physical Education, Health and Leisure Studies, National Cheng Kung University, Tainan, Taiwan; ^2^Department of Physical Education, National Kaohsiung Normal University, Kaohsiung, Taiwan; ^3^Biomedical Research Center, Institute of Experimental Endocrinology, Slovak Academy of Sciences, Bratislava, Slovakia; ^4^Faculty of Medicine, Institute of Pathological Physiology, Comenius University, Bratislava, Slovakia

**Keywords:** eye movement, antisaccade, prosaccade, high-intensity interval exercise, acute exercise

## Abstract

A wealth of evidence has shown that a single bout of aerobic exercise can facilitate executive function. However, none of current studies on this topic have addressed whether the magnitude of the acute-exercise benefit on executive function and oculomotor performance is influenced by different aerobic exercise modes. The present study was thus aimed toward an investigation of the acute effects of high-intensity interval exercise (HIIE) vs. moderate-intensity continuous exercise (MICE) on executive-related oculomotor performance in healthy late middle-aged and older adults. Using a within-subject design, twenty-two participants completed a single bout of 30 min of HIIE, MICE, or a non-exercise-intervention (REST) session in a counterbalanced order. The behavioral [e.g., reaction times (RTs), coefficient of variation (CV) of the RT], and oculomotor (e.g., saccade amplitude, saccade latency, and saccadic peak velocity) indices were measured when participants performed antisaccade and prosaccade tasks prior to and after an intervention mode. The results showed that a 30-min single-bout of HIIE and MICE interventions shortened the RTs in the antisaccade task, with the null effect on the CV of the RT in the late middle-aged and older adults. In terms of oculomotor metrics, although the two exercise modes could not modify the performance in terms of saccade amplitudes and saccade latencies, the participants’ saccadic peak velocities while performing the oculomotor paradigm were significantly altered only following an acute HIIE intervention. The present findings suggested that a 30-min single-bout of HIIE and MICE interventions modulated post-exercise antisaccade control on behavioral performance (e.g., RTs). Nevertheless, the HIIE relative MICE mode appears to be a more effective aerobic exercise in terms of oculomotor control (e.g., saccadic peak velocities) in late middle-aged and older adults.

## Introduction

Executive functions, referring to top-down cognitive processes, include three core components: response inhibition, cognitive flexibility, and working memory (Diamond, 2013). Such complex cognitive abilities enable an individual to independently perform complex, goal-directed, and self-serving activities (e.g., problem solving and inhibition of irrelevant processing) (Lezak et al., 2004). Impairment of executive functions is associated with poor functional achievement, lower ability to perform daily living tasks, and a more rapid progression to dementia (Royall et al., 2005; Tsai et al., 2018, 2019a). Therefore, executive dysfunctions affect the individual’s functional ability and increase the risk of cognitive decline in cognitively normal elderly individuals (Mirsky et al., 2011; Tsai et al., 2017b). Since the function of the frontostriatal network supporting executive functions decreases as a part of healthy aging (Buckner, 2004), and declines in executive function precede age-related memory loss (Blacker et al., 2007; Tsai et al., 2016a), determining how to circumvent executive dysfunction in individuals who are at increased risk for cognitive decline is an important issue.

It has been well established that a single bout of exercise can enhance executive functions in middle-aged and older adults, with behavioral performance being facilitated [e.g., shorter reaction times (RTs) and higher accuracy rates (AR)] following acute exercise when they perform cognitive tasks involving executive functioning (Kamijo et al., 2009; Barella et al., 2010; Johnson et al., 2016; Tsai et al., 2018; Formenti et al., 2020). In addition, in terms of cognitive electrophysiological performance, Kamijo et al. (2009) found that, as compared to the baseline session, event-related potential (ERP) P3 latency was significantly shorter following a bout of light or moderate aerobic exercise in older adults when performing a Flanker task. Tsai et al. (2018) also found that acute moderate aerobic exercise significantly enlarged ERP P3 amplitudes in middle-aged and older adults with mild cognitive impairment (MCI). However, some studies have found an inverted U-shaped relationship between acute-exercise intensity and cognitive performance (Kamijo et al., 2007) and have reported that the performance of executive function appears to be improved only with a single bout of moderate, not low or high, intensity exercise due to optimal acute-exercise-induced psychological and physiological arousal (Tomporowski, 2003; McMorris et al., 2010; Dietrich and Audiffren, 2011). In contrast, the “drive” hypothesis supports that the most beneficial effects on post-exercise executive improvements are elicited by heavy to near maximal levels of intensity (Chang et al., 2012) since regional cerebral blood flow and circulating neurotrophic factors levels increase with exercise intensity (i.e., engendering larger neurobiological magnitudes when individuals perform exercise with near maximal levels of intensity as compared to moderate intensity) (Knaepen et al., 2012; Tsai et al., 2014b). As a result, exercise intensity could be one of potential moderators of single-bout post-exercise executive benefits.

A robust body of literature has shown that a single bout of moderate intensity continuous exercise (MICE) can produce a transient facilitating effect on executive functions in various populations (Kamijo et al., 2007; Pontifex et al., 2009; Hung et al., 2013; Tsai et al., 2014a, 2016b, 2018; Hakansson et al., 2017; Formenti et al., 2020). High-intensity interval exercise (HIIE) is characterized by brief, intermittent bursts of intensive aerobic exercise interspersed with brief periods of low-intensity exercise or recovery (Laursen and Jenkins, 2002) and has recently proposed as a time-efficient alternative to traditional cardiorespiratory exercise (Alves et al., 2014; Tsai et al., 2021). Previous studies have reported that HIIE is more effective than MICE for increasing cardiovascular/metabolic health and exercise capacity in healthy individuals (Helgerud et al., 2007). The effectiveness of HIIE has also been demonstrated in older adults with various chronic diseases (Puhan et al., 2006; Tjønna et al., 2008; Angadi et al., 2015). Such a physical exercise mode has been promoted as a low-risk, practical, and time-efficient approach to optimizing health and well-being, thereby reducing the burden of chronic diseases associated with physical inactivity (Lucas et al., 2015). Although both of these aerobic exercise modes (i.e., HIIE and MICE) have been reported to modulate the impact on behavioral and cognitive electrophysiological performance, the potential effects are still somewhat equivocal at the moment (Tsukamoto et al., 2016; Schwarck et al., 2019; Tsai et al., 2021).

An antisaccadic task (i.e., looking mirror-symmetrical to a visual stimulus) highly correlates with neuropsychological measures of executive function and is a well-characterized measure of inhibitory control (Mirsky et al., 2011). The performance of antisaccade tasks declines with advancing age in normal elderly individuals (Peltsch et al., 2011). In addition, the antisaccade task is sensitive to frontal lobe processes that increase the risk of cognitive decline in older adults with or without incipient neurodegenerative progress (e.g., Alzheimer’s disease) (Mirsky et al., 2011). In contrast to prosaccades mediated *via* direct retinotopic projections within the superior colliculus (Wurtz and Albano, 1980), antisaccades is tightly regulated by frontoparietal executive networks involved in vector inversion and response inhibition (i.e., the suppression of a stimulus-driven prosaccade) that includes the frontal, supplementary, and parietal eye fields (Munoz and Everling, 2004; Everling and Johnston, 2013). As distinct from the prosaccadic task (i.e., saccade to a target’s veridical location), longer RTs, less ARs/increased directional errors, and more variable endpoints emerge when individuals perform an antisaccadic task (Gillen and Heath, 2014). Petrella et al. (2019) used prosaccadic and antisaccadic tasks to examine the effects of 10 min of continuous aerobic exercise at different intensities (i.e., moderate, heavy, and very heavy) on executive-related oculomotor performance in elderly adults. They found that antisaccade RTs, but not prosaccade RTs, were reduced across the continuum of moderate to very-heavy exercise intensities, indicating that the post-exercise benefits in antisaccade RTs did not reliably vary with exercise intensity (Petrella et al., 2019). Since cortical regions responsible for antisaccades show improved task-dependent prefrontal cortex activity following an exercise intervention (Colcombe et al., 2004), and impaired performance on the antisaccade task could serve as indices of potentially increased risk of cognitive decline in normal elderly individuals (Mirsky et al., 2011), the antisaccadic task is an ideal tool for detecting subtle changes in executive functions following a single bout of exercise in such a group (Petrella et al., 2019).

The age-associated declines in some cognitive domains (e.g., executive functions, memory, reasoning, and processing speed) have begun from middle age (i.e., 30 years old and onward) (Hedden and Gabrieli, 2004; Park and Reuter-Lorenz, 2009). Thus far, there have been no previous studies comparing the acute effects of different types and intensities of aerobic exercise (e.g., HIIE vs. MICE) on executive-related oculomotor control in individuals at risk for cognitive decline. Accordingly, the main purpose of the present study is to investigate the effects of a single bout of HIIE vs. MICE interventions on executive control in healthy late middle-aged and older adults when performing the saccade paradigm. Since a single-bout of aerobic exercise performed at a moderate-to-vigorous level of intensity produces a small but reliable cognitive benefit (e.g., executive control) (Lambourne and Tomporowski, 2010; Petrella et al., 2019), and the antisaccade task is a cognitive task modulated by the activity of prefrontal cortex, antisaccade planning processes could be facilitated by an exercise intervention (Samani and Heath, 2018). Therefore, it was hypothesized that an acute bout of 30 min of HIIE and MICE intervention would produce beneficial effects with regard to executive-related behavioral and oculomotor parameters in the exercise-intervention (i.e., HIIE and MICE) groups relative to those seen in the non-exercise-intervention (REST) group. In addition, an inverted U hypothesis regarding the acute exercise-and-cognitive performance relationship (i.e., optimal exercise-induced arousal elicited by acute moderate exercise intensity) was reported in a previous study (Kamijo et al., 2007). Accordingly, we also postulated that the facilitating effects on executive-related behavioral performance and oculomotor control induced by acute exercise would be more prominent in the MICE compared to HIIE intervention.

## Materials and Methods

### Participants

Twenty healthy community dwelling participants (10 males, ages 55–73) were recruited using convenience sampling *via* an informative flyer and word of mouth. This sample size was determined based on an *a priori* power analysis (alpha = 0.05, power = 0.80) to detect a medium-to-large effect (*d* = 0.06 ∼ 0.14) (Cohen, 1988) of acute MICE and HIIE on behavioral and oculomotor performance during the saccadic task as computed using G^∗^Power 3.1.9 (Faul et al., 2007), with the estimate of the minimum required sample size being 18. A health-screening questionnaire and a structured interview on previous medical history confirmed that the participants were free of significant orthopedic conditions, sensory impairment, neurological or psychiatric disorders, cerebrovascular and metabolic diseases, and substance abuse or addiction influencing central nervous system function that would limit the ability to exercise. All participants had normal or corrected-to-normal vision based on the minimum 20/20 standard, and were non-smokers and right-handed according to the Edinburgh Handedness Inventory. None of the participants exhibited any symptoms of depression, as measured by the Beck Depression Inventory II (DBI-II, all scored below 13), and identified objective cognitive impairments, as measured by the Mini-Mental State Examination (MMSE, all scored over 24) and the Montreal Cognitive Assessment (MoCA, all scored over 26). The participants’ baseline demographics and clinical characteristics are shown in Table 1. The participants gave written informed consent and visited the laboratory on four occasions to participate in the experimental protocol conducted in accordance with the Declaration of Helsinki and approved by the Institutional Ethics Committee of National Cheng Kung University.

**TABLE 1:** Demographic data.

Variables	Total (*n* = 20)
Age (years)	61.15 ± 4.43
Education (years)	13.75 ± 2.15
Height (cm)	162.85 ± 7.94
Weight (kg)	64.30 ± 7.59
BMI (kg/m^2^)	24.23 ± 2.27
Systolic BP (mmHg)	127.60 ± 19.54
Diastolic BP (mmHg)	77.35 ± 11.72
Resting heart rates (beats per minute)	69.70 ± 7.41
MMSE (scores)	29.00 ± 1.03
MoCA (scores)	27.85 ± 1.57
BDI-II (scores)	1.95 ± 2.31
IPAQ (MET-min/week)	1428.55 ± 743.66
Physical fitness	
Grip (kg)	30.57 ± 9.32
Arm curl (number)	37.45 ± 9.19
Chair stand (number)	22.20 ± 5.33
8-foot up-and-go (s)	5.13 ± 0.76
Back scratch (cm)	4.03 ± 4.11
Chair sit-and-reach (cm)	7.50 ± 5.57
Estimated VO_2__max_ (mL/kg/min)	35.57 ± 5.46

*BMI, body mass index; BP, blood pressure; MMSE, Mini-Mental Status Examination; MoCA, Montreal Cognitive Assessment; BDI-II, Beck Depression Inventory, 2nd edition.*

### Experimental Procedure

**FIGURE 1:**
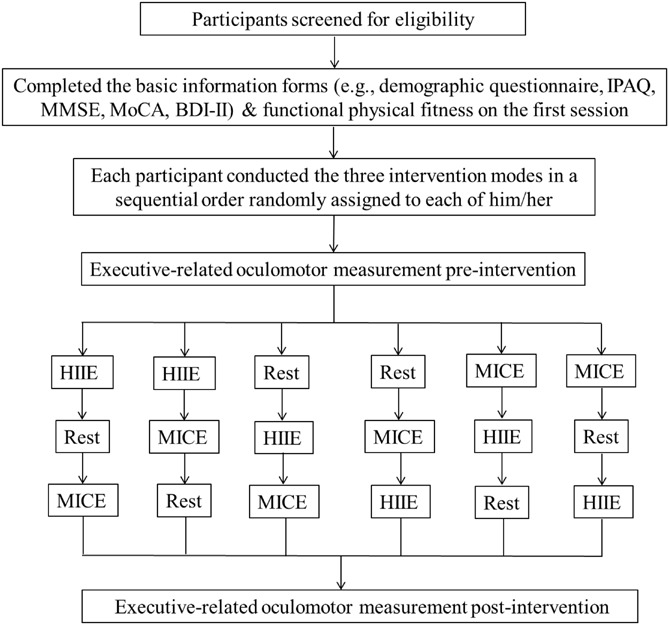
Flowchart of the study.

As illustrated in Figure 1, all participants visited the cognitive neurophysiology laboratory four times since a balanced within-subject cross-over design was adopted in the present study. On the first session, an informed consent form was signed prior to testing after the participants understood and agreed to participating in the experimental procedure. Then, the DBI-II, MMSE, MoCA, the basic information form (e.g., a medical history and demographic questionnaire), and a handedness inventory were administered. To lower potential exercise risks before the acute HIIE and MICE interventions, previous levels of physical activity, as assessed using the International Physical Activity Questionnaire (IPAQ), were used to calculate overall energy expenditure per week (MET-min/week) and resting heart rate, and a Physical Activity Readiness Questionnaire was also administered. The participants’ height and weight were measured to calculate their BMI. A certified fitness instructor then completed all assessments of senior functional physical fitness (Rikli and Jones, 2012) for each participant. The Rockport Fitness Walking Test (Kline et al., 1987) was used to estimate the participants’ cardiorespiratory fitness (i.e., VO_2__max_), in which the participant was required to walk one mile as quickly as possible, with the heart rate (HR) being continuously recorded using a Polar HR monitor (RX800CX, Finland).

For each of three (i.e., MICE, HIIE, or REST) intervention sessions, to control for circadian influences, the participants were required to come to the laboratory at about 8:30–9:30 am on three different days with an interval of 7 days between sessions. They were randomly allocated to a sequential order to conduct the three intervention sessions to minimize potential practice and mode of exercise effects (see Figure 1). Each participant was asked to get seven or 8 h of sleep before each intervention exercise session and was also asked refrain from participating in strenuous exercise and consuming alcohol for 24 h before their next arrival. All interventions were administered in an acoustically shielded room with a controlled temperature (23–25°C) and dimmed lights. At the beginning of each session, the participants’ body temperature (BT) and resting HR (HRrest) were measured, and then they were asked to be fitted with a Polar HR monitor (RX800CX, Finland) and to sit on a height adjustable chair in front of an IBM-compatible computer with their head placed comfortably in a head/chin rest, at a viewing distance of approximately 75 cm. After a practice session, the formal oculomotor test was immediately administered. All participants completed the saccadic tasks prior to and following a 30-min single bout of MICE, HIIE, or Rest intervention.

For the MICE and HIIE sessions, participants were introduced to a stationary adjustable bike and familiarized with the Borg’s rating of perceived exertion (RPE) scale (i.e., 6–20) to self-monitor and report individual effort perceptions to the experimenter every 1.5 min during exercise. The participant performed the two exercise modes on the bike for 30 min. MICE began with a 4 min warm-up, followed by 24 min of moderate-intensity exercise [50–55% Heart Rate Reserve (HRR)], and a 2 min cool-down. HIIE began with a 4 min warm-up, followed by 24 min of high-intensity intervals (1 min, 70–75% HRR) alternated with an active recovery period [2 min, target RPE = 9–11], and a 2 min cool-down. During the HIIE and MICE warm-up and cool-down, the participants’ RPE was set at levels ranging from 9–11. The exercise intensities of MICE and HIIE were monitored during which verbal encouragement was provided to facilitate exercise effort. After the acute HIIE and MICE interventions, HR was measured to ascertain it had returned to within 10% of the baseline (between 3 and 5 min following the cool-down). Then, the saccadic tasks test was carried out again. For the Rest session, the participants sat for 35 min and read magazines.

### The Saccadic Paradigm

The saccadic paradigm employed in the present study was adapted from Petrella et al.’s (2019) and Ranchet et al.’s (2017) studies. It consists of prosaccade and antisaccade tasks and has been shown to effectively assess the neuropsychological performance of executive function among middle-aged and elderly participants (Mirsky et al., 2011; Ranchet et al., 2017; Petrella et al., 2019). Each of the prosaccade and antisaccade tasks contained both gap and overlap trials to maximize task sensitivity (Briand et al., 2001). A trial commenced with a 3-s countdown followed 1,000 ms later by the appearance of a yellow fixation cross (1° in diameter, 135 cd/m^2^, 1,000 ms). In the gap condition, there was a gap interval of 200 ms, and then the eccentric white target stimuli (1° in diameter, 127 cd/m^2^) appeared randomly either to the left or right side and in the same horizontal meridian, in a proximal (10.5°) or distal (15.5°) position to prevent stereotyped responses (Gillen and Heath, 2014). In the overlap condition, when the eccentric target appeared, the fixation cross remained visible throughout the remainder of the trial. The target stimuli remained visible for 3,000 ms, after which the next trial started. Each of the prosaccade and antisaccade tasks were divided into eight blocks of 192 trials between runs, with the target location (i.e., left and right of fixation)/eccentricity (i.e., proximal and distal) and gap/overlap fixation conditions being randomly interleaved throughout each block of 24 trials. For the prosaccade task, participants were instructed to shift gaze from the central fixation point to a left or right target (i.e., saccade to the veridical target stimuli location). As soon as the participant looked at the target, he/she pressed a button of the computer keyboard to record the reaction time. For the antisaccade task, the same visual presentations were used as in the prosaccade task, in which the participants were asked to look away from the eccentric target to its mirror location (i.e., saccade mirror-symmetrical to the target stimuli location) and press a button of the computer keyboard. Prosaccade and antisaccade tasks were completed at each pre- and post-intervention session in separate, randomly ordered blocks.

### Oculomotor Recordings

The Tobii Pro X3-120 Eye Tracker (Tobii Technology, Inc., Stockholm, Sweden) was used to record the participants’ saccadic eye movement patterns at a sampling rate of 120 Hz. Saccade onset was determined by velocity values of greater than 30°/s, and saccade offset occurred when the velocity values were less than 30°/s for 20 ms. Given that the saccade metrics were strongly correlated with cognition (MacAskill et al., 2012; Connell et al., 2017), saccade latencies for correct trials were collected and determined as the duration of the interval from the appearance of an eccentric target to the onset of the first eye movement. Only latencies between 90 and 1,000 ms were analyzed (Chan et al., 2005). Saccade amplitude was defined as the angular distance the eye traveled during the movement, representing the saccade accuracy and spatial decision. Saccadic peak velocity was computed as the maximum eye velocity during the initial saccade.

### Data Processing and Statistical Analysis

Trials with missing data (e.g., a blink), RTs less than 100 ms (i.e., anticipatory saccades), and an saccadic amplitude less than 2° or greater than 2.5 standard deviations from the mean value, were excluded from the data analyses, thereby ruling out outliers that could skew the group means. Trials involving a directional error were also not included in the data analyses since such responses are mediated *via* planning processes that are distinct from individual’s directionally correct counterparts (DeSimone et al., 2014). The dependent variables included the RT (time from stimulus onset to button press of the computer keyboard), the coefficient of variation (CV) of the RT (standard deviation/mean × 100%), saccade amplitude, saccade latency, and saccadic peak velocity. Kolmogorov-Smirnov and Levene’s tests were used to confirm normality and homogeneity of variance assumptions, respectively. Since the main purpose in the present study aimed to investigate the acute effects of MICE and HIIE interventions on executive-related oculomotor performance in the participants when performing the prosaccade and antisaccade tasks and, importantly, previous studies demonstrated that acute aerobic exercise with moderate and intense instensities could not produce a significant main effect on the “target eccentricity by intervention mode” interaction in the young and older adults (Samani and Heath, 2018; Petrella et al., 2019), for increasing the statistical power of the analyses, dependent variables were thus submitted separately to a three-way mixed-model ANOVA, with the following factors: 3 (*Intervention mode*: MICE vs. HIIE vs. REST) × 2 (*Time*: pre-intervention vs. post-intervention) × 2 (*Task*: prosaccade vs. antisaccade). Posterior comparisons of the mean values with paired-sample multiple comparisons (adjusted using the Bonferroni correction) were conducted when the RM-ANOVAs revealed significant main effect interactions. Analyses employing the Greenhouse–Geisser correction with three or more within-subject levels were performed if a major violation of the assumption of sphericity was detected. For complementary use of significance testing, the effect size, partial η^2^ (η*_*p*_*^2^), was reported, with the effects of less than 0.08 considered small, 0.08–0.139 considered medium, and greater than 0.14 considered large. An alpha level of.05 was set for statistical significance.

## Results

Table 1 presents descriptive data for participants’ demographic characteristics. Briefly, the participants had a mean age of 61.15 ± 4.43 years and were free of depressive symptoms (BDI-II scores below 13) and cognitive impairment (MMSE scored over 24 and the MoCA scored over 26). Although the mean BMI values in the late middle-aged and older adults fell into the overweight category based on WHO criteria for Asian populations obtained from the Western Pacific Regional Office (Tsai et al., 2017a, 2019b), they exhibited enough physical activity levels (as seen from IPAQ data) (Sallis et al., 1985) and physical fitness (e.g., cardiorespiratory fitness) (Liskustyawati et al., 2020) to perform the acute HIIE and MICE interventions with lower potential risk factors.

In addition, the average RPE (MICE vs. HIIE: 10.95 ± 0.76 vs. 12.65 ± 0.67) and HRs (MICE vs. HIIE: 112.95 ± 1.50 vs. 129.10 ± 3.60 bpm) during the last 15 s of each minute were significantly lower during MICE than they were during HIIE (both *p*s < 0.001) in the present study.

### Behavioral Performance

#### Reaction Time

**FIGURE 2:**
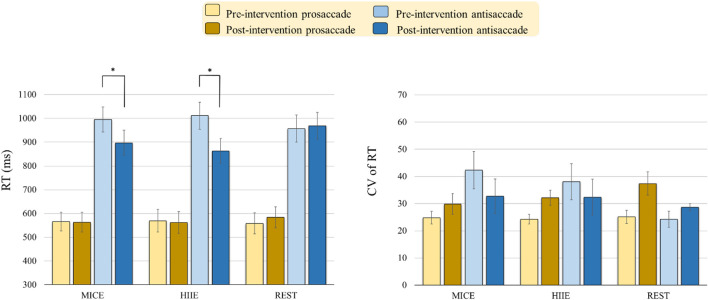
Behavioral performance [i.e., Reaction time (RT) and Coefficient of variation (CV) of RT] (mean ± SE) in the prosaccadic and antisaccadic tasks for the moderate intensity continuous exercise (MICE) and high-intensity interval exercise (HIIE) modes before and after a single bout of exercise, and one non-exercise-intervention (REST) before and after resting (*p < 0.05).

As illustrated in Figure 2, the RM-ANOVA on the RTs exhibited significant main effects of *Time* [*F*(1, 57) = 10.91, *p* = 0.002, η*_*p*_*^2^ = 0.16, power = 0.959] and *Task* [*F*(1, 57) = 177.55, *p* < 0.001, η*_*p*_*^2^ = 0.76, power = 1.000]. The *post hoc* analyses showed that the post-intervention RTs (739.83 ms) were shorter than the pre-intervention RTs (776.26 ms) across the three intervention modes and two tasks, and the RTs for the antisaccade task (948.99 ms) were longer than those for the prosaccade tasks (567.09 ms) across the three intervention modes and two time points. These main effects were superseded by the *Time* × *Intervention mode* [*F*(2, 57) = 6.82, *p* = 0.002, η*_*p*_*^2^ = 0.19, power = 0.952], *Time* × *Task* [*F*(1, 57) = 16.29, *p* < 0.001, η*_*p*_*^2^ = 0.22, power = 0.999], and *Time* × *Intervention mode* × *Task* [*F*(2,57) = 3.22, *p* = 0.047, η*_*p*_*^2^ = 0.10, power = 0.863] interactions. The *post hoc* analyses for the *Time* × *Intervention mode* × *Task* interaction indicated that the both MICE [*t*(19) = 2.20, *p* = 0.040] and HIIE [*t*(19) = 4.77, *p* < 0.001] modes produced significantly shorter RTs in the antisaccade task, but not in the prosaccade task, post- as compared to pre-intervention.

#### Coefficient of Variation of Reaction Time

The RM-ANOVA on the CV of the RT exhibited a significant main effect of *Intervention mode* × *Task* [*F*(2, 57) = 3.49, *p* = 0.037, η*_*p*_*^2^ = 0.11, power = 0.732]. The *post hoc* analyses indicated that only the value for the prosaccade task (31.29) was higher than it was for the antisaccade task (18.99) in the REST intervention mode. Neither significant main effects of *Intervention mode, Task*, and *Time* nor other significant interactions between the three factors (*p*s > 0.60 in all cases), were obtained.

### Oculomotor Performance

#### Saccade Amplitude

**FIGURE 3:**
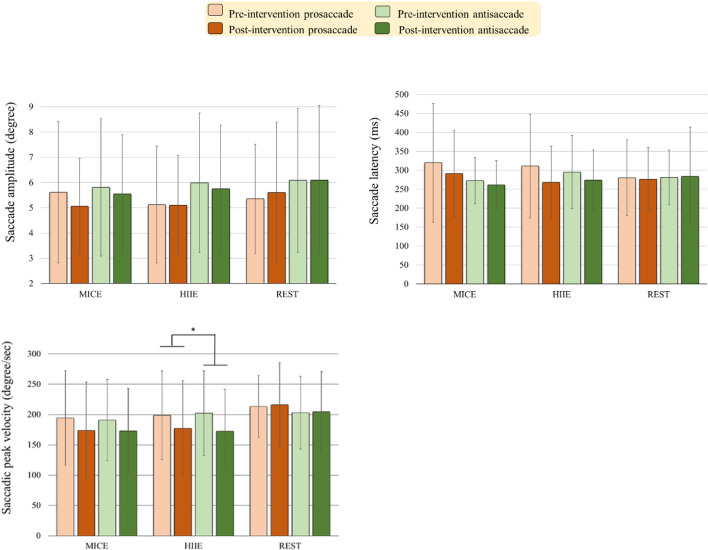
Oculomotor performance [i.e., Saccade amplitude, saccade latency, fixation duration, and saccadic peak velocity] (mean ± SD) in the prosaccadic and antisaccadic tasks for the moderate intensity continuous exercise (MICE) and high-intensity interval exercise (HIIE) modes before and after a single bout of exercise, and one non-exercise-intervention (REST) before and after resting (*p < 0.05).

As illustrated in Figure 3, the RM-ANOVA on the saccade amplitude exhibited significant main effects of *Task* [*F*(1, 57) = 5.52, *p* = 0.022, η*_*p*_*^2^ = 0.09]. The *post hoc* analyses indicated that saccade amplitudes were less for prosaccades (3.32°) than for antisaccades (3.88°) across the three intervention modes and across two time points. Neither significant main effects of *Intervention mode* and *Time* nor significant interactions between *Intervention mode, Task*, and *Time* (*p*s > 0.36 in all cases) were obtained.

#### Saccade Latency

The RM-ANOVA on the saccade latency exhibited significant main effects of *Time* [*F*(1, 57) = 5.79, *p* = 0.019, η*_*p*_*^2^ = 0.09]. The *post hoc* analyses indicated that saccade latencies were faster post- (275.89 ms) as compared to pre-intervention (293.36 ms) across the three intervention modes and across the two tasks. Neither significant main effects of *Intervention mode* and *Time* nor significant interactions between *Intervention mode, Task*, and *Time* (*p*s > 0.21 in all cases) were obtained.

#### Saccadic Peak Velocity

The RM-ANOVA on the saccadic peak velocity exhibited a significant main effect of *Time* [*F*(1, 57) = 9.21, *p* = 0.004, η*_*p*_*^2^ = 0.14, power = 0.928]. The *post hoc* analyses showed that the saccadic peak velocities were lower post-intervention (186.19 degree/s) as compared to pre-intervention (200.37 degree/s) across the three intervention modes and the two tasks. The main effect was superseded by the *Time* × *Intervention mode* [*F*(2, 57) = 3.24, *p* = 0.047, η*_*p*_*^2^ = 0.10, power = 0.683] interaction. The *post hoc* analyses for the *Time* × *Intervention mode* interaction indicated that the HIIE [*t*(19) = 4.06, *p* = 0.001] modes produced significantly shorter saccadic peak velocity across the two saccade tasks post- (174.94 degree/s) as compared to pre-intervention (200.45 degree/s). The MICE [*t*(19) = 1.85, *p* = 0.079] modes only approached significance in the saccadic peak velocity across the two saccade tasks post- (173.33 degree/s) as compared to pre-intervention (192.60 degree/s).

## Discussion

The objective of the current study was to examine the effects of acute HIIE and MICE interventions on behavioral and eye-movement performance in healthy late middle-aged and older adults when performing cognitive tasks involving executive-related oculomotor control. The main findings were that a 30-min single-bout of HIIE and MICE intervention modes could significantly shorten RTs in the antisaccade task in such a group, with the null effect on the CV of the RT. In terms of oculomotor performance, although the two exercise modes did not change the saccade amplitude and saccade latency performance, the saccadic peak velocity during the oculomotor tasks was significantly altered following an acute bout of HIIE, but not MICE, in the late middle-aged and older adults.

Separate parallel descending pathways involving the cerebral frontal and parietal cortex, basal ganglia, superior colliculus, and brainstem saccade generator differently control the generation of saccades, both visually guided (i.e., reflexive) and voluntary (Terao et al., 2013). The planning properties of prosaccades are operated with minimal top-down cortical control (Pierrot-Deseilligny et al., 1995) since spatial relations between stimulus and response are overlapped during the saccadic mode (Wurtz and Albano, 1980). In contrast, the antisaccade task is a task requiring the individual to inhibit a reflexive saccade toward the target (i.e., prosaccade) and instead generate a voluntary-triggered saccade toward a mirror position without a visual target stimulus (Terao et al., 2013). In the present study, longer RTs for the antisaccade task were shown as compared to the prosaccade task when the participants performed the saccadic paradigm, reflecting that such a cognitive task is time-consuming since it is associated with high-level executive demands related to inhibiting a stimulus-driven prosaccade (i.e., pre-potent response), transforming a target’s coordinate, and decoupling the spatial relations between the stimulus and the response (i.e., vector inversion) (Samani and Heath, 2018; Petrella et al., 2019). As such, the present findings demonstrated that the oculomotor paradigm adopted in the present study provides a viable framework for investigating acute-exercise-related changes to executive function in late middle-aged and older adults.

Similar to Tsai et al.’s (2014a) study exploring various neurocognitive indices after acute aerobic exercise in young adults with different cardiorespiratory fitness levels when performing a visuospatial attention task and finding the facilitated RTs and cognitive electrophysiological effects (e.g., event-related potential P3 amplitudes and Contingent Negative Variation), the participants in the present study showed significantly shorter RTs following a single bout of HIIE and MICE when performing the executive-related oculomotor paradigm. Both previous and recent studies demonstrate that a reliable post-exercise improvement in executive functions is supported by acute-exercise-based increases in regional cerebral blood flow to executive-related cortical structures and, importantly and specifically, by enhancing arousal, cortical efficiency, and attentional allocation in adults when performing cognitive tasks involving inhibitory control (Dietrich and Audiffren, 2011; Tsai et al., 2014a, 2018, 2021; Verburgh et al., 2014; Formenti et al., 2020). However, it is worth noting that shorter RTs post- as compared to pre-exercise interventions were only found in the antisaccade task, but not in the prosaccade one in the present study. This finding is in line with a previous work by Petrella et al. (2019) reporting that 10-min of single-bout aerobic exercise with different exercise intensities did not alter prosaccade metrics in healthy older adults. There are two plausible mechanisms to account for the pattern in the findings. It is likely that, given the reflexive nature of prosaccades (e.g., humans complete upward of 150,000 prosaccades per day) (Robinson, 1981), neural correlates of prosaccades supported by the midbrain are refractory to a single bout of exercise intervention (Petrella et al., 2019). Another possibility is that prosaccades operate independently of the vector inversion and response suppression executive demands (Pierrot-Deseilligny et al., 1995). However, since the antisaccade task is linked to increased activation of executive-related frontoparietal networks that support maintenance of high-level task rules (Ettinger et al., 2008; Everling and Johnston, 2013; Weiler et al., 2015), the present results support the premise that a single bout of 30 min of HIIE and MICE are effective in terms of improving the brain’s executive control in late middle-aged and older adults (Tsai et al., 2021). It is noteworthy that, relative to Petrella et al.’s (2019) study, pre- and post-oculomotor assessments were performed in separate sessions interleaved by a non-exercise (REST) condition in the present study, and non-significant changes in RTs in this intervention mode were observed. The present finding demonstrated that the improved RTs in the antisaccade task after a single bout of HIIE and MICE could not be attributed to practice effects and that executive-control-related performance (e.g., inhibitory control) benefits could be elicited *via* the two exercise modes in late middle-aged and older adults.

Although Petrella et al. (2019) found that a 10-min single bout of continuous aerobic exercise with moderate, heavy, and very heavy-heavy exercise intensities could not alter the oculomotor performance (e.g., eye-movement time, and saccade amplitude, and variability in the horizontal movement direction), and the post-exercise facilitation in antisaccade RTs did not reliably vary with exercise intensity in older adults, changes in the magnitude of the executive-related oculomotor control on saccadic peak velocity influenced by different exercise intensities was observed in the late middle-aged and older adults in the present study, with significant decrements in the peak velocity of saccades emerging following a 30-min single bout of HIIE, but not MICE. One possible explanation for these disparate findings is that the present finding regarding the altered saccadic peak velocity following the acute HIIE in part supports the previous studies reporting that short duration exercise sessions (less than 20 min) produce a null effect on executive control performance, whereas durations greater than 20 min produce a significant effect (Lambourne and Tomporowski, 2010; Chang et al., 2012). In addition, there is a relationship between brain catecholamines (e.g., dopamine and norepinephrine) and saccadic control (Allman et al., 2012). Indeed, the levels of peripheral norepinephrine are linked to saccadic peak velocity (Connell et al., 2017). Acute exercise could cause changes in central neurotransmitters (i.e., increased levels of norepinephrine) (Peake et al., 2014; McMorris, 2016), which could further reduce saccadic peak velocity (Connell et al., 2017). Since the acute HIIE relative to MICE intervention appeared to induce higher levels of norepinephrine (Peake et al., 2014), it is plausible that a significant alteration in saccadic peak velocity was only observed after a single bout of 30 min of HIIE, but not MICE, in the present study. Until now, the acute HIIE relative to MICE effects on executive benefits have appeared to be divergent. For example, Schwarck et al. (2019) found that the null-effect on cognitive response to an acute bout of MICE and HIIE was possibly due to a small sample size. However, a larger improvement in the trail-making task assessing cognitive flexibility was exhibited following MICE as compared to HIIE in the identified responder (Schwarck et al., 2019). In contrast, Tsukamoto et al. (2016) compared the effects of acute HIIE and MICE on executive function in young adults when performing the color-words Stroop task and found that, although the two aerobic exercise modes could improve cognitive performance, the post-exercise executive benefit during 30-min of recovery was sustained in the HIIE but not in the MICE mode. In the present study, although acute HIIE and MICE interventions equally improved the RTs in the antisaccade task, the former relative to the latter exercise mode appears to induce more oculomotor control performance related to saccadic peak velocity. However, it is still worth noting that the alteration in the oculomotor index approached the borderline of significance at 30-min post-exercise following MICE. Future research efforts should continue to address this unclear finding (e.g., exploring what effect aerobic training has on the norepinephrine response to exercise of the different intensities). Importantly, norepinephrine is associated with cognitive function (Holland et al., 2021), with an age-dependent reduction in such a molecular level being exhibited with poor contextual learning and memory (Dang et al., 2014). Also, increasing synaptic norepinephrine activity could improve response inhibition (Liu et al., 2015). It is conceivable that alterations to norepinephrine *via* acute HIIE and MICE interventions could retard age-related declines in executive control. In addition, the saccadic peak velocity seems to be a sensitive non-invasive index by which to observe neurocognitive changes.

Nevertheless, our results did not confirm significant changes in other saccade parameters (e.g., saccade amplitude and latency), which are considered interdependent with saccadic peak velocity. Saccadic amplitude is reported to be influenced by an interplay of processes in the basal ganglia, frontal cortex, and brainstem (Terao et al., 2016). In addition, the amplitude of the saccade made during visual scanning is associated with the function of basal ganglia (Machado and Rafal, 2004). In accordance with the findings of previous studies (Samani and Heath, 2018; Petrella et al., 2019) reporting that 10-min single-bouts of aerobic exercise at moderate to vigorous intensities did not significantly alter the saccade amplitudes in young and older adults, these oculomotor indices were also not significantly improved following a 30-min single-bout of HIIE and MICE in the late middle-aged and older adults in the present study. One plausible reason for the null effect on this oculomotor metric is that the specification of antisaccade amplitudes is mediated through retinotopic motor maps in the superior colliculus (Wurtz and Albano, 1980) in which the brain subcortical structure is considered to be refractory to exercise-based modulations (Colcombe and Kramer, 2003). However, there was a trend toward lower saccade amplitudes following the acute HIIE and MICE interventions in the present study since the reduced amplitude for the saccade task may be explained by the inhibition of the superior colliculus *via* the basal ganglia (Machado and Rafal, 2004), the two exercise modes could be sufficient to inhibit excessive neural impulses in this brain area.

Similarly, a significant difference in saccade latency was also not exhibited after a single bout of 30 min of HIIE and MICE in the late middle-aged and older adults in this study, which could be attributed to that, as mentioned above, saccade latency is correlated with the magnitude of response in the superior colliculus (Neggers et al., 2005). Until now, there has been no previous study exploring the effect of acute exercise on saccade latency. Nevertheless, Di Russo et al. (2003) found that elite shooters have faster saccadic latency to targets than controls in saccade tasks under standard (i.e., performing a visually guided saccade toward a target as fast as possible as the prosaccade condition in the present study) and distracter (i.e., only saccading toward the red, not the green, stimulus) conditions. In fact, the saccadic latency performance has been found to be associated with attentional levels (Di Russo et al., 2003) when a saccadic program toward a predictable direction is partially or completely prepared before the appearance of eccentric targets (Pare and Munoz, 1996). The facilitating effect that acute exercise has in terms of eliciting more attentional resource allocation (e.g., larger brain event-related potential P3 amplitude following a single bout of 30 min of exercise) has been demonstrated in many previous studies (Tsai et al., 2014a, 2016b, 2018). As such, faster saccade latency following a single bout of HIIE and MICE could be expected when the late middle-aged and older adults performed the oculomotor paradigm. However, there was only a decreasing, but not significant, trend on the saccade latencies observed post- relative to pre-acute-exercise in the present study. How the effect of the saccadic motor preparation coded at the retinotopic level through chronic exercise (Di Russo et al., 2003) could be significantly induced following an acute exercise intervention is worth investigating in further experiment.

There are limitations to the oculomotor approach that must be addressed. First, the posteriori power data of the statistical analyses for the behavioral and oculomotor results were 0.732–1.000 and 0.683–0.928, respectively. Although η*_*p*_*^2^ was also included to provide a measure of effect size in the present study, the small sample size could still be a limitation. A confirmatory study in the future is required to determine whether some null effects translates into significant improvements following a single bout of acute MICE and HIIE protocols in a larger sample. Second, a significant correlation between dynamic saccade latency and visual acuity of saccadic eye movement has been reported (Kohmura et al., 2008). Accordingly, the accurate discrimination of a moving target at high speed could be influenced by the early start of saccadic eye movement. Because of age-related diabetes, macular degeneration, and cataracts, middle-aged and older adults frequently lose visual acuity as they age (Munoz et al., 2000). When performing oculomotor tasks, these individuals have to execute a fast saccade to an eccentric target stimuli. Although normal or corrected-to-normal vision was one of inclusion criteria in the present study, the non-significant finding regarding the saccade latencies after the acute HIIE and MICE interventions could not be avoided due to potential optic nerve degeneration/eye diseases in the middle-aged and older adults. Further investigation of this issue before the oculomotor experiment is warranted. Third, since a lingering inhibition that will elicit the unidirectional prosaccade switch cost (i.e., delayed planning of a subsequent prosaccade) will be engendered by the completion of an antisaccade (Weiler and Heath, 2014), future research efforts using randomly interleaved pro- and antisaccade trials into one block (e.g., a task-switching paradigm) will be necessary to confirm and better understand the specific mechanism contributing to improved executive-related oculomotor control in the antisaccade task elicited by the acute HIIE/MICE intervention, as well as an investigation of the performance benefits/costs following correct and incorrect antisaccade trials (Samani and Heath, 2018).

## Conclusion and Perspectives

Impaired performance on executive function tests precedes cognitive decline in the normal elderly population and conversion to Alzheimer disease in older adults with MCI (Blacker et al., 2007; Tsai et al., 2016a, 2018, 2019a). In the present study, a post-exercise antisaccade, but not prosaccade, benefit on behavioral performance (e.g., RTs) was observed in the late middle-aged and older adults when performing the oculomotor paradigm. In addition, the peak velocity of the first saccadic eye movement was only significantly altered after the HIIE, but not the MICE, intervention. These findings imply that a single bout of HIIE and MICE could produce a short-term “boost” to executive-related cognitive control in late middle-aged and older adults, supporting that the two acute aerobic exercise interventions improved task-specific activity within the frontoparietal networks supporting antisaccades. Nevertheless, the HIIE relative to the MICE mode appears to be a more effective alternative to the alteration of oculomotor control (i.e., saccadic peak velocity) in late middle-aged and older adults, possibly due to acute-exercise-induced in brain neurotransmitters (e.g., norepinephrine). In addition, it is worth noting that, given the deficits in executive functions and saccadic eye movement shown in the patients with neurodegenerative diseases (e.g., Alzheimer’s disease and Parkinson’s disease) (Tsai et al., 2018, 2019a; Chen et al., 2021; Lage et al., 2021), HIIE could be one of safe and preventive intervention strategies in lowering their declining cognitive functions and oculomotor control in clinical and medical settings.

## Data Availability Statement

The raw data supporting the conclusion of this article will be made available by the authors, without undue reservation.

## Ethics Statement

The studies involving human participants were reviewed and approved by Institutional Ethics Committee of National Cheng Kung University. The patients/participants provided their written informed consent to participate in this study.

## Author Contributions

C-LT designed the study, wrote the protocol and the first draft of the manuscript. Y-CC and T-CW collected and analyzed the data. C-YP, JU, and BU reviewed and edited the manuscript. All authors contributed to the article and approved the submitted version.

## Conflict of Interest

The authors declare that the research was conducted in the absence of any commercial or financial relationships that could be construed as a potential conflict of interest.

## Publisher’s Note

All claims expressed in this article are solely those of the authors and do not necessarily represent those of their affiliated organizations, or those of the publisher, the editors and the reviewers. Any product that may be evaluated in this article, or claim that may be made by its manufacturer, is not guaranteed or endorsed by the publisher.
